# Prescribing LAIs: from completing the first injection to going steady

**DOI:** 10.1017/S1092852925100588

**Published:** 2025-10-29

**Authors:** Andrew J. Cutler, Meghan M. Grady

**Affiliations:** 1 Department of Psychiatry, SUNY Upstate Medical University, Syracuse, NY, USA; 2 Neuroscience Education Institute, Malvern, PA, USA

**Keywords:** Long-acting injectable (LAI), antipsychotic, dosing, pharmacokinetics, maintenance treatment

## Abstract

Non-adherence and even partial adherence to antipsychotic treatment can increase the risk of relapse in patients with schizophrenia. One strategy to improve adherence is through the use of long-acting injectable (LAI) antipsychotics. Multiple LAI antipsychotic options are available, which differ in terms of their formulation, administration, initiation, and maintenance dosing schedule. This article provides a practical guide to the conversion from oral to LAI antipsychotic treatment for the available LAI formulations as well as evidence-based principles for maintenance treatment.

## Long-acting injectable antipsychotics reduce risk of relapse

Non-adherence and even partial adherence can increase the risk of relapse in patients with schizophrenia.^1^^,^^2^ Relapse in turn carries multiple potential consequences, including brain tissue loss, rehospitalization, treatment resistance, functional disability, suicide, arrests/incarceration, and homelessness.^3^^–^^5^ A proven strategy to prevent relapse is the use of long-acting injectable (LAI) antipsychotics, which can provide steady-state therapeutic drug levels with an injection schedule ranging from 2 to 26 weeks, depending on the particular formulation. Studies have shown that patients prescribed an LAI formulation are more likely to be adherent than those prescribed an oral formulation.^6^^–^^8^ Indeed, the majority of data show that the risk of relapse and rehospitalization is reduced with LAI antipsychotics compared to oral formulations.^8^^–^^12^

Although not every antipsychotic has an LAI formulation as an option, there are formulations available for fluphenazine, haloperidol, risperidone, paliperidone, aripiprazole, and olanzapine; in the cases of risperidone, paliperidone, and aripiprazole, multiple LAI formulations exist (Table 1). Each LAI has a unique profile in terms of pharmacokinetics, administration factors, initiation strategy, and maintenance dosing schedule. This article provides a practical guide to the conversion from oral to LAI antipsychotic treatment for the available LAI formulations as well as evidence-based principles for maintenance treatment.

**Table 1.** Initiation and maintenance dosing for long-acting injectable antipsychotics

LAI	Initial dosing	Maintenance dosing	Injection type and site
Aripiprazole lauroxil (Aristada)^13^^,^^14^	(1) Any Aristada dose with oral aripiprazole coverage for 21 days or (2) Single-dose 675-mg injection of Aristada Initio in combination with a 30-mg oral dose; first maintenance Aristada injection can be administered on the same day as the single-dose injection or up to 10 days later	441 mg, 662 mg, or 882 mg/4 weeks or 882 mg/6 weeks or 1064 mg/8 weeks Max: 882 mg/4 weeks	Intramuscular deltoid (441 mg dose only) or gluteal (all doses)
Aripiprazole monohydrate 1-month (Maintena)^15^	(1) Two separate 400-mg injections of Maintena with a single oral dose of 20 mg aripiprazole or (2) One initial injection of 400 mg Maintena and oral antipsychotic coverage for 14 days	300–400 mg/4 weeks; max 400 mg/4 weeks	Intramuscular gluteal
Aripiprazole monohydrate 2-month (Asimtufii)^16^	For patients currently receiving oral antipsychotic: (1) One injection of 960 mg Asimtufii, one injection of 400 mg Maintena, and a single oral dose of 20 mg aripiprazole or (2) One initial injection of 960 mg Asimtufii and oral antipsychotic coverage for 14 days For patients currently receiving Maintena: 960-mg Asimtufii injection in place of the next-scheduled Maintena injection	720–960 mg/8 weeks; max: 960 mg/8 weeks	Intramuscular gluteal
Fluphenazine decanoate (Prolixin Decanoate)^17^^–^^19^	Per plasma level data: 3 weekly loading injections of 50 mg (lower doses for first episode or less severely ill); first maintenance dose 2 weeks after the third loading dose Per package insert: 12.5 to 25 mg as the initiation dose; subsequent injections and dosing intervals based on the patient’s response	12.5–75 mg/2 weeks Max: 75 mg/week	Intramuscular or subcutaneous
Haloperidol decanoate (Haldol Decanoate)^17^^,^^20^^–^^22^	Per plasma level data: 3 weekly loading injections of 10 times the oral daily dose; may require oral coverage (half dose) for the first week; first maintenance dose 2 weeks after the third loading dose Per package insert: initial dose of 10–20 times the oral daily dose; if conversion requires more than 100 mg as an initial dose, it should be administered in 2 injections (100 mg initially and the balance in 3–7 days)	20 times the oral daily dose during the early phase of treatment: 25–300 mg/4 weeks Max: 300 mg/2 weeks	Intramuscular
Olanzapine pamoate (Zyprexa Relprevv)^23^	Loading during the initial 8 weeks; dose and schedule are based on correspondence to oral olanzapine doses	150–300 mg/2 weeks 300–405 mg/4 weeks Max: 300 mg/2 weeks	Intramuscular gluteal
Paliperidone palmitate 1-month (Invega Sustenna and Erzofri)^24^^,^^25^	Loading doses of 234 mg on day 1 and 156 mg on day 8 (Invega Sustenna) or 351 mg on day 1 (Erzofri); maintenance dose should start 4 weeks after the second loading injection; dosing is based on correspondence to oral paliperidone doses	39, 78, 117, 156, or 234 mg/4 weeks Max: 234 mg/4 weeks	Intramuscular (deltoid at initiation, then either deltoid or gluteal)
Paliperidone palmitate 3-month (Invega Trinza)^26^	Initiate 3-month LAI when the next 1-month LAI injection is scheduled after at least 4 months of 1-month injections; dosing is based on the previous 1-month injection dose	273, 410, 546, or 819 mg/12 weeks Max: 819 mg/12 weeks	Intramuscular deltoid or gluteal
Paliperidone palmitate 6-month (Invega Hafyera)^27^	Initiate 6-month LAI when the next 1-month or 3-month LAI injection is scheduled after at least 4 months of 1-month injections or after at least one 3-month cycle of the 3-month injection; dosing is based on the previous 1-month or 3-month injection dose	1092 or 1560 mg/26 weeks Max: 1560 mg/26 weeks	Intramuscular gluteal only
Risperidone microspheres (Consta and Rykindo)^28^^,^ ^29^	Loading is not possible, necessitating oral coverage for 7 days (Rykindo) or 21–28 days (Consta)	12.5–50 mg/2 weeks Max: 50 mg/2 weeks	Intramuscular deltoid (Consta only) or gluteal
Risperidone subcutaneous (Uzedy)^30^	No loading or oral supplementation required	50–125 mg/4 weeks or 100–250 mg/8 weeks; max 250 mg/8 weeks	Subcutaneous abdomen or upper arm

## Converting to LAIs: general principles

### Choosing the LAI dose

The dose equivalence of oral to LAI antipsychotic formulations is well defined for some but not all of the LAI options. Choosing the appropriate LAI dose is best accomplished by understanding the current extent of medication exposure—in other words, by obtaining plasma antipsychotic levels.^17^ Prior to switching to an LAI, plasma antipsychotic levels should be obtained as 12-hour morning trough values for oral medications at steady state.^17^ If antipsychotic plasma levels are low, repeating the trough plasma level can help determine whether this is due to an adherence issue or a pharmacokinetic one: fluctuations of more than 30% typically represent poor adherence, assuming the levels were drawn at comparable times.^17^

### Initial dosing

When converting from an oral antipsychotic to an LAI, for most formulations, one must either adequately load the dose or provide oral supplementation. A loading dose is the dose required to immediately achieve a plasma concentration that is equivalent to the steady-state concentration.^17^ Dose loading is possible for many but not all of the available LAI formulations; when not available, oral supplementation is required. The failure to adequately load the dose or provide oral supplementation can lead to subtherapeutic antipsychotic plasma levels for weeks or months.

### Maintenance dosing

Obtaining antipsychotic plasma levels can be beneficial not only for initiating the dosing of an LAI, but also during maintenance treatment. Because plasma antipsychotic levels increase gradually over time, dose requirements may eventually decrease, making it valuable, when possible, to obtain periodic plasma levels to prevent unnecessary plasma level creep. The appropriate time to get a blood level for patients receiving an LAI is the morning of the day they will receive their next injection; however, levels can be obtained up to 72 hours prior to the next injection.^17^

## Converting to haloperidol decanoate (Haldol Decanoate)

The response threshold for haloperidol is typically 2 to 5 ng/mL, while plasma levels greater than 18 ng/mL are generally not well tolerated.^17^^,^^31^ Haloperidol decanoate can be loaded; the recommended loading dose in the literature is 10 times the oral daily dose, given weekly for the first 3 weeks (Table 1).^20^^,^^21^ Discontinuation of oral antipsychotic can begin immediately if adequate loading is pursued; however, the time to maximum concentration ranges from 3 to 9 days, and oral coverage (one-half the oral dose) may still be necessary for the first week for some patients.^17^

Steady state is reached after 4 weeks with loading. The terminal half-life with multiple dosing is 21 days; therefore, the maintenance dosing schedule for haloperidol decanoate is every 4 weeks.^22^ The maintenance dose during the early phase of treatment is 20 times the oral daily dose and should start 2 weeks after the last loading injection.^17^ Depending on the appropriate maintenance dose, some patients may require a different dosing schedule. Single injection volumes greater than 3 ml (300 mg) are not tolerated,^22^ so patients who require higher doses typically receive the monthly dose as split injections every 2 weeks.

Trough plasma levels should be checked every 2–3 months during the first year of treatment with haloperidol decanoate. In the event of level creep, the maintenance dose may need to be adjusted downward. According to the package insert, the usual maintenance dose is 10–15 times the previous daily dose.^22^

## Converting to fluphenazine decanoate (Prolixin Decanoate)

For fluphenazine decanoate, the response threshold is 1 ng/mL. Plasma levels greater than 2 to 3 ng/mL may not be well tolerated, with 4 ng/mL considered the “point of futility.”^17^ With a single dose, the time to maximum concentration is only 0.3 to 1.5 days, after which the concentration drops steeply.^32^ This early peak relative to other LAIs may make fluphenazine decanoate preferable in acute situations, but it also carries the risk of drug-induced parkinsonism or akathisia in the first 48 hours.^17^ In addition, the steep drop in levels can lead to relapse. Without loading, the time to steady state is more than 12 weeks; therefore, when converting to fluphenazine decanoate, one should initiate treatment with weekly loading injections.^18^

The formula for converting patients from oral to long-acting fluphenazine is not as well established as for some other LAIs, and the best way to determine the loading schedule is by obtaining a plasma level. Studies have shown that 3 weekly loading injections of 50 mg will yield a plasma level above the response threshold of 1 ng/mL.^18^ Some patients, such as those with first-episode or less severe illness, may receive lower doses.^17^ The package insert for fluphenazine states that, for most patients, 12.5 to 25 mg can be used as the initiation dose, with subsequent injections and dosing intervals based on the patient’s response.^19^

The first maintenance dose should be administered 2 weeks after the last loading injection. The usual maintenance dose is 12.5 to 75 mg every 2 weeks and should be guided by plasma levels. 25 mg/2 weeks is associated with trough plasma levels of 1–1.2 ng/mL.^33^^,^^34^

Single injections cannot exceed 3 ml due to tolerability, so higher doses must be administered weekly; the maximum dose is 75 mg/week.^17^ As with other LAI antipsychotics, trough plasma levels should be checked every few months to avoid level creep.

## Converting to risperidone formulations

There are three available LAI formulations of risperidone, two of which are administered intramuscularly and one subcutaneous. The established plasma level response threshold for risperidone is 15 ng/mL. The point of futility is 112 ng/mL; however, there is no available dosage form of risperidone LAI that approaches that level (Table 2).^17^ This means that patients who are taking higher doses of oral risperidone (more than 5–6 mg/day) may not be good candidates for the LAI formulations, since more than one injection would be required.

**Table 2.** Dose equivalence for risperidone LAI formulations^17^^,^^28^^–^^30^^,^^35^^–^^38^

Daily oral risperidone dose (mg)	Average steady-state plasma level (ng/mL)	Microspheres dose every 2 weeks (Consta or Rykindo) (mg)	Subcutaneous dose once monthly (Uzedy) (mg)	Subcutaneous dose once every 2 months (Uzedy) (mg)
2	14	25	50	100
3	21	37.5	75	150
4	28	50	100	200
5	35	50	125	250

### Risperidone microspheres: Consta and Rykindo

Risperidone microspheres are administered intramuscularly. The lag time in active release means that maximum concentration is not achieved until 14–17 days for Rykindo and 21 days for Consta.^32^ However, the terminal half-life with multiple doses is 3 to 6 days, which is why these LAIs need to be dosed every 2 weeks.^28^^,^^29^ Steady state is achieved after 4 injections, at 6 weeks.

Risperidone microspheres cannot be loaded, so oral supplementation is required to maintain therapeutic plasma levels (Table 1). Consta requires 21 days of oral overlap, while Rykindo requires 7 days of oral coverage.^28^^,^^29^ Plasma concentrations can be estimated as approximately 7 times the oral dose, which can in turn be used to predict the appropriate long-acting dose (Table 2).^17^ For example, a 2-mg oral dose should correspond to an active moiety plasma level of approximately 14 ng/mL, which in turn corresponds to 25 mg of risperidone microspheres. However, this is an average and can vary by individual patient, so obtaining plasma levels is ideal when deciding on the maintenance dose of the LAI formulation.

The usual maintenance dose range is 12.5–50 mg every 2 weeks, with 50 mg per vial as the highest available dosing option.^28^^,^^29^ Changes in blood levels due to dosage changes (or missed dose) are not apparent for 3–4 weeks; if dose adjustments are needed after a patient has started risperidone microspheres, titration should occur at intervals of no less than 4 weeks.^17^ If a dose is missed by 2 or more weeks, then oral coverage while reinitiating injections may be necessary.

### Risperidone subcutaneous: Uzedy

Although there are two approved subcutaneous risperidone formulations, one (Perseris) is no longer being marketed.^30^^,^^39^ With the copolymer technology utilized for subcutaneous risperidone, the delivery system is applied as a liquid and hardens upon contact with bodily fluids.^17^^,^^30^^,^^39^ This allows for an initial release of the active drug that reaches therapeutic plasma levels within 24 hours, thus obviating the need for oral coverage or a second loading injection, followed by controlled release for up to 2 months.^17^^,^^30^^,^^39^

The injection volumes are smaller than with the intramuscular formulations (typically less than 1 ml); this allows for less-invasive injection sites (abdominal or upper arm rather than gluteal or deltoid).^17^^,^^30^ There are two absorption peaks: the first occurs within 6–24 hours due to an initial release of the active drug during the depot formation process.^35^^–^^38^ The second occurs 8–14 days after the injection, with similar magnitude and at levels that approach steady state (Table 1).^35^^–^^38^ Steady state is reached in 2 months.

Uzedy can be administered every month (50–125 mg) or every two months (100–250 mg) in either the abdomen or the upper arm.^30^ The average exposure values over the dosing period are comparable for once-monthly and once every two months administration at corresponding doses.

## Converting to paliperidone palmitate formulations

Paliperidone palmitate exists as a 1-month, a 3-month, and a 6-month formulation (Table 1). The 1-month formulation is an option for patients who are switching from oral medication, from a different LAI, or who are not on active medication.^24^^,^^25^ The 3-month formulation is only to be used for patients who have already received adequate treatment with 1-month paliperidone palmitate for at least 4 months.^26^ The 6-month formulation is only for patients who have received adequate treatment with the 1-month formulation for at least 4 months or the 3-month formulation for at least one 3-month cycle.^27^

### 1-month paliperidone palmitate (Invega Sustenna and Erzofri)

For patients switching from oral medication or who are not on active medication, the 1-month formulation of paliperidone palmitate can be loaded, with a standard loading schedule of 234 mg on day 1 and 156 mg on day 8, plus or minus 2 days (Sustenna).^24^ In 2024, another formulation of 1-month paliperidone palmitate (Erzofri) was approved, which has a single initiation loading dose of 351 mg.^25^ The initiation dose for either 1-month formulation must be administered in the deltoid muscle, as deltoid absorption is 28% greater than gluteal absorption. The maintenance dose should start 4 weeks after the second loading injection, but the dosing window is flexible and can vary by plus or minus 1 week.^24^^,^^25^ Patients who are switching from an LAI antipsychotic do not require the 1-week dose initiation schedule and instead can receive the first injection of paliperidone palmitate in place of their next scheduled depot injection.

The maintenance dose of 1-month paliperidone palmitate is determined based on the oral dose (Table 3), although ideally plasma levels would be obtained. The therapeutic threshold is 20 ng/ml; point of utility is not well established for paliperidone, and instead, the risperidone level is generally used as the best guide.^17^^,^^41^

**Table 3.** Dose equivalence for paliperidone formulations^23^^–^^25^^,^^38^^,^^39^

Daily oral risperidone dose (mg)	Daily oral paliperidone dose (mg)	1-month Invega Sustenna or Erzofri dose (mg)	3-month Invega Trinza dose (mg)	6-month Invega Hafyera dose (mg)
~1	3	39		
~2	3	78	273	
~3	6	117	410	
~4	9	156	546	1,092
~6	12	234	819	1,560

### Three-month paliperidone palmitate (Invega Trinza)

Three-month paliperidone palmitate is only for patients who have received adequate treatment with 1-month paliperidone for at least 4 months.^26^ The last 2 doses of 1-month paliperidone should ideally be the same dosage strength, so that a consistent maintenance dose is established prior to starting the 3-month formulation. The injection of 3-month paliperidone palmitate is given in place of the next-scheduled 1-month injection, with dosing based on the previous 1-month injection dose (Table 3). The dosing window is also flexible for the 3-month formulation, and it can be given up to 2 weeks before or after the 3-month time period. Dose adjustments can be made every 3 months if needed.

If a dose is missed for 4–9 months, a reinitiation schedule with the 1-month formulation must be followed (Table 4).^26^ If more than 9 months has passed since the last dose, treatment must be reinitiated with the 1-month formulation according to its prescribing information;^24^^,^^25^ patients can convert to the 3-month formulation once they have been adequately treated with the 1-month LAI for at least 4 months.

**Table 4.** Reinitiation schedule for 3-month paliperidone palmitate (4–9 months since last dose)^26^

Last dose of Invega Trinza (mg)	Day 1	Day 8	1 month after day 8
273	78 mg Invega Sustenna/Erzofri (deltoid)	78 mg Invega Sustenna/Erzofri (deltoid)	273 mg Invega Trinza (deltoid or gluteal)
410	117 mg Invega Sustenna/Erzofri (deltoid)	117 mg Invega Sustenna/Erzofri (deltoid)	410 mg Invega Trinza (deltoid or gluteal)
546	156 mg Invega Sustenna/Erzofri (deltoid)	156 mg Invega Sustenna/Erzofri (deltoid)	546 mg Invega Trinza (deltoid or gluteal)
819	156 mg Invega Sustenna/Erzofri (deltoid)	156 mg Invega Sustenna/Erzofri (deltoid)	819 mg Invega Trinza (deltoid or gluteal)

### Six-month paliperidone palmitate (Invega Hafyera)

The injection of the 6-month formulation of paliperidone palmitate is given in place of the next-scheduled 1-month or 3-month injection, with dosing based on the previous 1-month or 3-month product (Table 3).^27^ When switching from the 1-month LAI, the last two 1-month doses should be the same dosage strength so that a consistent maintenance dose is established prior to starting the 6-month formulation. When switching from the 3-month LAI, the 6-month injection can be given up to 2 weeks before or after the next-scheduled 3-month dose. Dose adjustments can be made every 6 months if needed; response to an adjusted dose may not be apparent for multiple months.

Patients taking the 6-month formulation can receive their next injection up to 2 weeks before or 3 weeks after the next-scheduled 6-month dose. If more than 6 months and 3 weeks has passed since the last dose, reinitiation with 1-month Sustenna is necessary (Table 5).^27^

**Table 5.** Reinitiation schedule for 6-month paliperidone palmitate (at least 6 months and 3 weeks since last dose)^27^

Up to 8 months since last dose of Invega Hafyera			
Last dose of Invega Hafyera (mg)	Day 1		1 month after day 1
1,092	156 mg Invega Sustenna/Erzofri (deltoid)		1,092 mg Invega Hafyera (gluteal)
1,560	234 mg Invega Sustenna/Erzofri (deltoid)		1,560 mg Invega Hafyera (gluteal)

8–11 months since last dose of Invega Hafyera			
Last dose of Invega Hafyera (mg)	Day 1	Day 8	1 Month After Day 8
1,092	156 mg Invega Sustenna/Erzofri (deltoid)	156 mg Invega Sustenna/Erzofri (deltoid)	1,092 mg Invega Hafyera (gluteal)
1,560	156 mg Invega Sustenna/Erzofri (deltoid)	156 mg Invega Sustenna/Erzofri (deltoid)	1,560 mg Invega Hafyera (gluteal)

More than 11 months since last dose of Invega Hafyera			
Reinitiate treatment with Invega Sustenna or Erzofri according to its prescribing information; Invega Hafyera can be used after the patient has been adequately treated with Invega Sustenna or Erzofri for at least 4 months			

## Converting to aripiprazole formulations

Aripiprazole is available in more than one LAI formulation, which differ in terms of their formulation, kinetics, and initiation strategies. Response threshold for aripiprazole is 110 ng/ml, which corresponds to 10 mg/day of oral aripiprazole, and point of futility is 500 ng/ml, which is the plasma level associated with 100% dopamine 2 (D2) receptor occupancy.^17^

### Aripiprazole monohydrate: Maintena and Asimtufii

Aripiprazole monohydrate is available in a 1-month (Maintena) and a 2-month (Asimtufii) formulation. Aripiprazole monohydrate is poorly soluble, resulting in slow and prolonged dissolution and absorption, with maximum concentration achieved after about a week with Maintena and after 28 days with Asimtufii.^17^ Until recently, it could not be loaded and thus required oral coverage for the first 14 days; however, it is now an option to use a 2-injection loading strategy in lieu of oral coverage (Table 1).^15^^,^^16^

The 1-day initiation of Maintena requires two separate 400-mg injections with a single oral dose of 20 mg aripiprazole. The other option is a 14-day initiation, which requires an initial injection of 400 mg along with 14 days of overlapping oral antipsychotic. The maintenance dosing schedule is typically 300 to 400 mg every 4 weeks.^15^ The maximum dose, 400 mg, is equivalent to 20 mg of oral aripiprazole, which generally corresponds to a plasma level in the 200s.^15^^,^^17^ Steady state is achieved after 4 monthly injections. In the event of a missed dose (more than 5 weeks between doses if steady state is not yet achieved and more than 6 weeks between doses once steady state is achieved), it is necessary to restart treatment with either the 1-day initiation or the 14-day initiation.^15^

Asimtufii can be initiated in patients receiving oral antipsychotic. The 1-day initiation requires one injection of 960 mg Asimtufii, one injection of 400 mg Maintena, and a single oral dose of 20 mg aripiprazole. Alternatively, the 14-day initiation requires one initial injection of 960 mg Asimtufii and oral antipsychotic coverage for 14 days. When converting from Maintena to Asimtufii, a 960-mg Asimtufii injection should be administered in place of the next-scheduled Maintena injection.^16^

The maintenance dose of Asimtufii is 720 mg or 960 mg every 2 months. The dosing window is flexible, and the next injection can be given up to 2 weeks before or after the 2-month time period.^16^ If more than 14 weeks has elapsed between injections, it is necessary to restart treatment with either the 1-day initiation or the 14-day initiation.^16^

Aripiprazole monohydrate cannot be used with strong cytochrome 450 (CYP) 3A4 inducers and requires dose adjustment in the presence of CYP2D6 and CYP3A4 inhibitors, as well as in patients who are poor CYP2D6 metabolizers (Table 6).^15^^,^^16^

**Table 6.** Dose adjustments for aripiprazole monohydrate due to CYP interactions^15^^,^^16^

	Adjusted dose for patients taking:		
	300 mg Maintena	400 mg Maintena	Asimtufii
Poor 2D6 metabolizers	N/A	300 mg	N/A
Patients taking strong 2D6 or 3A4 inhibitors	200 mg	300 mg	720 mg
Poor 2D6 metabolizers taking concomitant 3A4 inhibitors	N/A	200 mg	Avoid use
Patients taking 2D6 AND 3A4 inhibitors	160 mg	200 mg	Avoid use
Patients taking 3A4 inducers	Avoid use	Avoid use	Avoid use

### Aripiprazole lauroxil: Aristada and Aristada Initio

Aripiprazole lauroxil is a prodrug formulation, which allows for the formation of crystals that dissolve slowly. Once the prodrug is released from the crystal, it is immediately cleaved by hydrolysis, releasing active aripiprazole that reaches maximum concentration after 44 to 50 days, with four monthly injections required to reach steady state.^13^ There are two options for how to initiate treatment: (1) oral coverage for the first 21 days or (2) use of the 675-mg single-dose initiation injection in combination with a 30-mg dose of oral aripiprazole (Table 1).^14^ The first maintenance aripiprazole lauroxil injection can be administered on the same day as the single-dose injection or up to 10 days later. One should avoid injecting both the single-dose injection and maintenance-dose injection into the same deltoid or gluteal muscle.^14^

The dosing equivalence of oral aripiprazole to aripiprazole lauroxil is well defined (Table 7).^13^

**Table 7.** Dose equivalence for aripiprazole lauroxil^13^

Daily oral dose (mg)	Aristada dose	Intramuscular site
10	441 mg per month	Deltoid or gluteal
15	662 mg per month OR 882 mg every 6 weeks OR 1064 mg every 2 months	Gluteal
20–30	882 mg per month	Gluteal

Dose adjustments are needed in the presence of CYP2D6 inhibitors, CYP3A4 inhibitors, and CYP3A4 inducers (Table 8).^13^ However, dose adjustments are not possible for the single-dose injection, so this treatment initiation option should be avoided in patients who are known CYP2D6 poor metabolizers or who are taking strong CYP3A4 inhibitors, strong CYP2D6 inhibitors, or strong CYP3A4 inducers.^14^

**Table 8.** Dose adjustments for aripiprazole lauroxil due to CYP interactions^13^

	Adjusted dose for patients taking:			
	441 mg Aristada	662 mg Aristada	882 mg Aristada	1064 mg Aristada
Poor 2D6 metabolizers	N/A	N/A	N/A	N/A
Patients taking strong 2D6 or 3A4 inhibitors	N/A	441 mg	662 mg	882 mg
Poor 2D6 metabolizers taking concomitant 3A4 inhibitors	N/A	441 mg	441 mg	441 mg
Patients taking 2D6 AND 3A4 inhibitors	N/A	Avoid use	Avoid use	Avoid use
Patients taking 3A4 inducers	662 mg	N/A	N/A	N/A

## Converting to olanzapine pamoate (Zyprexa Relprevv)

For olanzapine pamoate, the response threshold is generally 20 ng/mL, although much higher levels may be tolerated; the point of futility is 150 ng/mL.^17^ The time to maximum concentration is 3 to 4 days, and the time to steady state is 3 months. The dose should be loaded for the first 8 weeks, with the specific dosage determined based on the previous oral dose (Table 9).^23^ Maintenance dosing can be every 2 weeks or every 4 weeks.

**Table 9.** Dosing equivalence for olanzapine formulations^23^

Daily oral dose (mg)	Zyprexa Relprevv dose: first 8 weeks	Zyprexa Relprevv dose: after 8 weeks
10	210 mg every 2 weeks OR 405 mg every 4 weeks	150 mg every 2 weeks OR 300 mg every 4 weeks
15	300 mg every 2 weeks	210 mg every 2 weeks OR 405 mg every 4 weeks
20	300 mg every 2 weeks	300 mg every 2 weeks

The main limitation to using olanzapine pamoate is that it has a Risk Evaluation and Mitigation Strategy (REMS) program that requires 3-hour post-injection monitoring due to the rare risk of post-injection delirium/sedation syndrome from vascular breach.^17^^,^^23^

## Summary

LAIs can reduce the risk of relapse and rehospitalization in patients with schizophrenia and should be discussed with patients as potential treatment options. Multiple first- and second-generation antipsychotics are available in LAI formulations, with others in development; in some cases, there are multiple LAI formulations for a particular active moiety. Each LAI has a unique profile in terms of formulation, administration, initiation, and maintenance injection schedule, with pharmacokinetics playing a vital role in these profiles.

## CNS SPECTRUMS

### CME Review Article

#### Prescribing LAIs: From Completing the First Injection to Going Steady

*This CME activity is provided by HMP Education and Neuroscience Education Institute (NEI).*

### CME/CE Information

**Target Audience:** This activity has been developed for the healthcare team or an individual prescriber specializing in mental health. All other healthcare team members interested in psychopharmacology are welcome for advanced study.

**Learning Objectives:** After completing this educational activity, you should be better able to:

- Facilitate a smooth transition when converting from oral to long-acting injectable (LAI) antipsychotics
- Apply evidence-based principles to the maintenance dosing of LAI antipsychotics

**Accreditation:** In support of improving patient care, this activity has been planned and implemented by HMP Education and Neuroscience Education Institute (NEI). HMP Education is jointly accredited by the Accreditation Council for Continuing Medical Education (ACCME), the Accreditation Council for Pharmacy Education (ACPE), and the American Nurses Credentialing Center (ANCC), to provide continuing education for the healthcare team.

**Activity Overview:** This activity is best supported via a computer or device with current versions of the following browsers: Mozilla Firefox, Google Chrome, or Safari. A PDF reader is required for print publications. A post-test score of 70% or higher is required to receive CME/CE credit.
**Estimated Time to Complete:** 1 hour.

Continuing Education credit will be available for three (3) years from the publication date of the associated article. Please visit https://nei.global/cnsspectrums2025 for additional information and to access the CE activity.

**NEI maintains a record of participation for six (6) years.*

#### Instructions for Optional Posttest and CME Credit

1. Read the article
2. Successfully complete the posttest at https://nei.global/CNS/LAI-05
3. Print your certificate

Questions? Email: customerservice@neiglobal.com.

**Credit Designations:** The following are being offered for this activity:

- **Physician: ACCME *AMA PRA Category 1 Credits*™**
  - HMP Education designates this enduring material for a maximum of 1.00 *AMA PRA Category 1 Credit*™. Physicians should claim only the credit commensurate with the extent of their participation in the activity.
- **Nurse: ANCC contact hours**
  - This continuing nursing education activity awards 1.00 contact hour. Provider approved by the California Board of Registered Nursing, Provider #18006 for 1.00 contact hour.
- **Nurse Practitioner: ACCME *AMA PRA Category 1 Credit*™**
  - American Academy of Nurse Practitioners National Certification Program accepts *AMA PRA Category 1 Credits*™ from organizations accredited by the ACCME.
  - The content in this activity pertaining to pharmacology is worth 1.00 continuing education hour of pharmacotherapeutics.
- **Pharmacy: ACPE application-based contact hours**
  - This internet enduring, knowledge-based activity has been approved for a maximum of 1.00 contact hour (.10 CEU).
  - *The* official *record of credit will be in the CPE Monitor system. Following ACPE Policy, NEI and HMP Education must transmit your claim to CPE Monitor within 60 days from the date you complete this CPE activity and are unable to report your claimed credit after this 60-day period. Ensure your profile includes your DOB and NABP ID.*
- **Physician Associate/Assistant: AAPA Category 1 CME credits**
- HMP Education has been authorized by the American Academy of PAs (AAPA) to award AAPA Category 1 CME credits for activities planned in accordance with the AAPA CME Criteria. This internet enduring activity is designated for 1.00 AAPA Category 1 credit. Approval is valid until February 11, 2028. PAs should only claim credit commensurate with the extent of their participation.
- **Psychology: APA CE credits**
- Continuing Education (CE) credits for psychologists are provided through the co-sponsorship of the American Psychological Association (APA) Office of Continuing Education in Psychology (CEP). The APA CEP Office maintains responsibility for the content of the programs. This activity awards 1.00 CE Credit.
- **Social Work: ASWB-ACE CE credits**
  - As a Jointly Accredited Organization, HMP Education is approved to offer social work continuing education by the Association of Social Work Boards (ASWB) Approved Continuing Education (ACE) program. Organizations, not individual courses, are approved under this program. Regulatory boards are the final authority on courses accepted for continuing education credit. Social workers completing this internet enduring course receive 1.00 general continuing education credit.
- **Non-Physician Member of the Healthcare Team: Certificate of Participation**
  - HMP Education awards hours of participation (consistent with the designated number of *AMA PRA Category 1 Credit*™) to a participant who successfully completes this educational activity.

**Peer Review:** The content was peer-reviewed by an MD, LFAPA specializing in psychiatry, forensics, and addiction—to ensure the scientific accuracy and medical relevance of information presented and its independence from commercial bias. NEI and HMP Education take responsibility for the content, quality, and scientific integrity of this CME/CE activity.

**Disclosures:** All individuals in a position to influence or control content are required to disclose any relevant financial relationships. Any relevant financial relationships were mitigated prior to the activity being planned, developed, or presented.
***Faculty Author / Presenter***

**Andrew J. Cutler, MD**

*Clinical Associate Professor of Psychiatry, SUNY Upstate Medical University, and Chief Medical Officer, Neuroscience Education Institute, Malvern, PA*

Consultant/Advisor: AbbVie, Acadia, Alfasigma, Alkermes, Anavex Life Sciences, Arrivo BioVentures, Autobahn Therapeutics, Axsome, Biogen, Biohaven, Boehringer Ingelheim, Brii Biosciences, Bristol Myers Squibb, Cerevel, Cognitive Research Corporation, Corium, Delpor, Evolution Research Group, 4M Therapeutics, Intra-Cellular Therapies, Janssen/J&J Innovative Medicine, Jazz Pharma, Karuna, LivoNova, Lundbeck, Luye Pharma, MapLight Therapeutics, MedAvante-ProPhase, Mentavi, Neumora, Neurocrine, Neuroscience Education Institute, NeuroSigma, Noven, Otsuka, PaxMedica, Relmada, Sage Therapeutics, Sirtsei Pharmaceuticals, Supernus, Teva, Thynk, Tris Pharma, Vanda Pharmaceuticals, VistaGen

Speakers Bureau: AbbVie, Alfasigma, Alkermes, Axsome, Boehringer Ingelheim, Bristol Myers Squibb, Corium, Intra-Cellular Therapies, J&J, Lundbeck, Neurocrine, Noven, Otsuka, Supernus, Teva, Tris Pharma, Vanda Pharmaceuticals

Stock Options: 4M Therapeutics

Board Member (Data Safety Monitoring Board): Alar Pharma, COMPASS Pathways, Freedom Biosciences, Pain Therapeutics

**Meghan Grady, BA**

*EVP, Content and Strategy, Neuroscience Education Institute, an HMP Global Company, Malvern, PA*

No financial relationships to disclose.

The remaining Planning Committee members, Content Editors, Peer Reviewer, and NEI planners/staff have no financial relationships to disclose. NEI and HMP Education planners and staff include Caroline O’Brien, MS, Ali Holladay, Moriah Carswell, Andrea Zimmerman, EdD, CHCP, Brielle Calleo, and Bahgwan Bahroo, MD, LFAPA.

**Disclosure of Off-Label Use:** This educational activity may include discussion of unlabeled and/or investigational uses of agents that are not currently labeled for such use by the FDA. Please consult the product prescribing information for full disclosure of labeled uses.

**Cultural Linguistic Competency and Implicit Bias:** A variety of resources addressing cultural and linguistic competencies and strategies for understanding and reducing implicit bias can be found in this handout—download me.

Accessibility Statement

For questions regarding this educational activity, or to cancel your account, please email customerservice@neiglobal.com.

**Support:** This activity is supported by an unrestricted educational grant from Alkermes, Inc., Teva Pharmaceuticals, Johnson & Johnson Innovative Medicine, and Otsuka America Pharmaceutical, Inc.
